# Effect of *Nigella sativa* on general health and immune system in young healthy volunteers; a randomized, placebo-controlled, double-blinded clinical trial

**DOI:** 10.12688/f1000research.73524.1

**Published:** 2021-11-25

**Authors:** Ayad Salem, Abdullah Bamosa, Mohammed Alam, Saleh Alshuraim, Hamad Alyalak, Abdulrahman Alagga, Faisal Tarabzouni, Omar Alisa, Hussein Sabit, Ashfaq Mohsin, Mohammad Shaikh, Abdulaziz Farea, Thamer Alshammari, Obeid Obeid

**Affiliations:** 1 Department of Physiology, college of Medicine, Imam Abdulrahman bin Faisal university, Dammam, Saudi Arabia; 2 College of Medicine, imam Abdulrahman bin Faisal university, Dammam, Saudi Arabia; 3 Department of Genetics, Institute of Medical Research and Consultations, Imam Abdulrahman bin Faisal university, Dammam, Saudi Arabia; 4 Department of Pharmaceutics, College of Clinical Pharmacy, Imam Abdulrahman bin Faisal university, Dammam, Saudi Arabia; 5 Department of Family and Community Medcine, College of Medicine, imam abdulrahman bin faisal univeristy, Dammam, Saudi Arabia; 6 Department of Microbiology, College of Medicine, imam abdulrahman bin faisal univeristy, Dammam, Saudi Arabia

**Keywords:** Nigella sativa, black seed, immunoglobulins, CD, immune system, immunity, gene expression

## Abstract

Nigella sativa ( *N. sativa*) is traditionally used as an immune enhancer in different communities. The aim of this study was to evaluate the effect of *N. sativa* on immunity related parameters in young healthy subjects. This study was a double blind, randomized, placebo controlled clinical trial. Fifty-two healthy subjects (48 male and 4 female) 18-25 years old were enrolled in the study. They were randomly divided into four groups; the first received charcoal capsules and served as controls and the other three received 0.5, 1 g, and 2 g of powdered *N. sativa* capsules, respectively. Two blood samples were obtained from all participant, before initiation of the trial and at the end of the four weeks intervention. One sample was used for routine health screening by evaluating liver and renal functions as well as complete blood count and differential. The second sample was used to measure certain cytokines including; IL-1, IL-4, IL-6, IL-10, and TNF. A third and fourth samples were obtained from the last cohort of subjects before and after treatment; the third was used for measuring immunoglobulins and CD profile and the fourth for evaluating certain gene expressions (INF-γ, NF-κ-B, TNF-α, IL-1β, IL-13, IL-8, and IL-6). Only 1 g dose of *N. sativa* produced a significant elevation in total lymphocyte count, CD3+ and CD4+ counts. One gram *N. sativa* increased the absolute lymphocyte count from 1850±0.24 to 2170±0.26 (p=0.008), CD3+ from 1184.4±75.60 to 1424±114.51 (p=0.009), and CD4+ from 665.6±141.66 to 841±143.36 (p=0.002).  This elevation in T cells was lost by increasing the dose of *N. sativa* to 2g. The rest of the parameters were not changed significantly in all doses.

The results show a promising immunopotentiation effect of *N. sativa* by elevating helper T cells and the optimum dose for young age group seems to be 1 g.

## Introduction

*Nigella sativa* is one of the most used herbal medicines worldwide nowadays. It has been used for more than 2000 years as a natural remedy for various illnesses. Research has documented its therapeutic potential as an antimicrobial, anti-inflammatory, antioxidant, antidiabetic, antihypertensive, antitumor, and immunomodulatory agent. ^1^^,^^2^

The immune system consists of multiple linked networks of cells, proteins, and lymphoid organs to provide protection against millions of microbes and infections. The immune system includes the innate immunity and the adaptive immunity, where the innate immunity provides an immediate protection to the body, and its effect is merely the same in different individuals, while the adaptive immunity, takes more time to develop, but it is more specific and fatal to invasive pathogens. ^3^ The immunomodulatory effect of *N. sativa* has been studied extensively on both innate and adaptive immunity as well as related messengers and mediators. ^4^^–^^6^

Several *in vivo* studies showed significant effects of *N. sativa* on immunity, autoimmune diseases, and toxicity. A study demonstrated that aqueous extract of *N. sativa* showed a significant increase (62.3% ± 6.4%) in the natural killer (NK) cytotoxic cell activity against YAC-1 cells after 1 week of oral administration in a 10-week old BALB/c female mice. ^7^ Another *in vitro* study on the effect of thymoquinone (TQ) on immunity showed that TQ injected directly to cells in low concentrations (10, 2.5 or 0.62 μg mL ^−1^), increased the survival of activated T-cells, CD8+ T-cells ability to generate IFN-γ, which indicates that TQ may be beneficial against infectious diseases and enhancing immunity. ^8^ *N. sativa* supplementation to newly evolved crossbred laying hens at levels of 4% or 5% positively enhanced immunity against Newcastle disease virus. ^9^ In another study on Newcastle virus vaccinated broilers, *N. sativa* supplementation in three doses (5, 10 and 20 g kg ^−1^) for 42 days, increased anti-bodies against Newcastle virus significantly on day 35. ^10^ A diet supplemented with 40 g kg ^−1^ *N. sativa* fed to broiler chicks improved anti-body production against both Newcastle virus and infectious bursal disease. ^11^ The phagocytic index and rate were significantly higher in STZ-diabetic hamsters treated with *N. sativa* oil (NSO) at a dose of 400 mg·kg ^−1^ for 4 weeks compared with untreated diabetic animals, as demonstrated by fluorescence microscopy. ^12^ *N. sativa* extract has stimulatory effect on cellular immunity, *in vitro*, by increasing the proliferative capacity of T lymphocytes and splenocytes and response to a different mitogens of the human peripheral blood mononuclear cells (PBMC). ^13^ NSO was shown to possess a protective role against vitamin A hypervitaminosis. Rats treated with 800 mg·kg ^−1^ NSO orally showed higher serum levels of IgG and IgM than either the control or those with high doses of vitamin A. ^14^ Gestational diabetes rats showed improvement in their offspring immune status, after oral antenatal feeding with 20 mg kg ^−1^ TQ, by reversing the decreased levels of IL-2, T-cell reproduction, and improving both circulating and thymus homing T-cells proliferation. ^15^

The above literature shows a very promising immunomodulating effect of *N. sativa.* However, the immunopotentiation effect of this miracle plant has not been investigated in normal humans. Hence this study was designed to evaluate the impact of different doses of *N. sativa* on the immune system in young healthy humans.

## Methods

This is a placebo-controlled, double blinded, randomized clinical trial. The study was conducted on healthy male and female students studying in Imam Abdulrahman bin Faisal University (IAU), Dammam, Saudi Arabia. Blood extraction was carried out in the main campus University Family Medicine Center. Students received the intervention for one month and were divided into four groups; three were given different doses of black seed and the fourth was given charcoal and served as control.

### Participants

The participants were students enrolled in different colleges in IAU. Subjects were randomly divided into four groups; 30 participants were allocated to each group through computer generated randomization table. The sample size was determined based on our previous clinical trials using *N. sativa.* ^16^^,^^17^ The first group was the control group (placebo) and they were given 162 mg of activated charcoal capsules, second group received 500 mg *N. sativa* capsules, third and fourth group received 1 and 2 g *N. sativa* capsules, respectively.

### Inclusion criteria

1. Healthy IAU students
2. Age between 18 and 25 years
3. BMI = 18.5–29.9 kg m ^−2^

### Exclusion criteria

1. Subjects with any acute or chronic illness (unless acute illness occurred during the study)
2. Subjects with abnormalities in the basic laboratory investigations
3. Participants with less than 90% compliance

## Material

Ethiopian *N. sativa*, bought from the local market, was cleaned, ground and assembled into 500-mg capsules, in the pharmaceutics laboratory in the College of Clinical Pharmacy at IAU. Activated charcoal capsules (162 mg) similar in size and color to the capsules of *N. sativa* (Arkopharma Pharmaceutical Laboratories Carros, France) were used as placebo. The placebo capsules were given in the same bottles as the *N. sativa* capsules. Each participant was given enough capsules for the period of 4 weeks. Bottles in each group were coded by the technical staff in the laboratory to achieve the double blindness in the study. The code was unmasked at the end of the study after statistical analysis of all data.

### Study protocol

After applying the inclusion and exclusion criteria mentioned above, recruited participants were given a full explanation of the study and required procedures and those who agreed to join, signed written consent. Subjects were recruited in three cohorts on Sundays from 08:00–10:00 h, in the period of February and March, 2020. Full history and physical examination were obtained from each participant to rule out any acute or chronic illnesses. Two blood samples were collected from all participants, in the Family and Community Medicine (FAMCO) Center in the IAU campus, before initiation of the study and at the end of the four-week study duration. The first sample was assessed in the center’s laboratory for basic tests which included complete blood count (CBC), renal function test (RFT) and liver function test (LFT) to assure the general health of the participant. The second sample was assessed in the microbiology laboratory in the College of Medicine at IAU to determine baseline cytokines level by enzyme-linked immunosorbent assay (ELISA). A third and fourth blood sample was collected from the last cohort of participants, before and after intervention. The third sample was used to measure CD profile by flow cytometric kits as well as immunoglobulins (IgG, IgM). The fourth blood sample was collected in heparin tubes to evaluate the gene expression profile of IFN-γ, NF-κB, TNF-α, IL-1β, IL-13, IL-8, and IL-6. Participants were followed daily by telephone calls for the whole study period (4 weeks) to ensure taking the capsules. ELISA kits for IL-1, IL-4, IL-6, IL-10, and TNF were bought from Origin company, USA, and the cytokines levels were measured according to the manufacturer recommendation. CD profile was measured using flow cytometric kits (TBNK kit, BD biosciences, USA).

The study has been approved by the university ethical board under the reference (IRB-2020-UGS-01-032) and was registered in ISRCTN registry (ISRCTN14150499, 16/11/2020, https://doi.org/10.1186/ISRCTN14150499).

### Gene expression analysis

We have evaluated the changes in the expression of different immunity-related genes listed in the table below, before and after intervention.

**Table:** 

Gene name	Forward	Reverse
IL1β	GCTGAGAAGGGCTTCATTCCA	TGCTGTGTCCCTAACCACAA
IL8	GCAGAGCTGTGCCTGTTGAT	TCCTAACACCTGGAACTTTCCTAAA
IFN-γ	AATGCTTTGCAAGACCCTCG	ATCCTCTGTTTGTGCTCTTTCCT
IL6	CCTGGCGATAACCAATTTTCCC	TTCCCCCACACCAAGTTGAG
IL13	TGACCCCTCGGTGTCCC	TGTGAGAGGGTGGGGGATG
TNF-α	AACCATTCTCCTTCTCCCCAA	CCCAAACCCAAACCCAGAATTAG
NF-κB	TCCATGTTGCTGGAGAGTCAG	AGGGGCCTGTTCATTCTAAGT

StepOne Plus thermal cycler was used in this study, and the thermal profile was as follows: 94°C for 2 min as pre-PCR and 95°C for 30 sec, 62°C for 45 sec, and 72°C for 45 sec for 35 cycles. Followed by 72°C for 10 min as post-PCR step. The 2 ^−ΔΔCt^ equation was used to analyze the fold change.

### Statistical analysis

Statistical analysis was performed based on intension to treat protocol using the Statistical Package of Social Science (SPSS) version 16 (RRID:SCR_019096); JASP (RRID:SCR_015823) is an open-access alternative. Data is presented as mean ± SD (standard deviation). In each group, readings were compared to their corresponding baseline values using Student’s t-test for paired data. Results in the four groups were compared using ANOVA. A *P*-value <0.05 was considered as significant.

## Results

**Figure 1.**
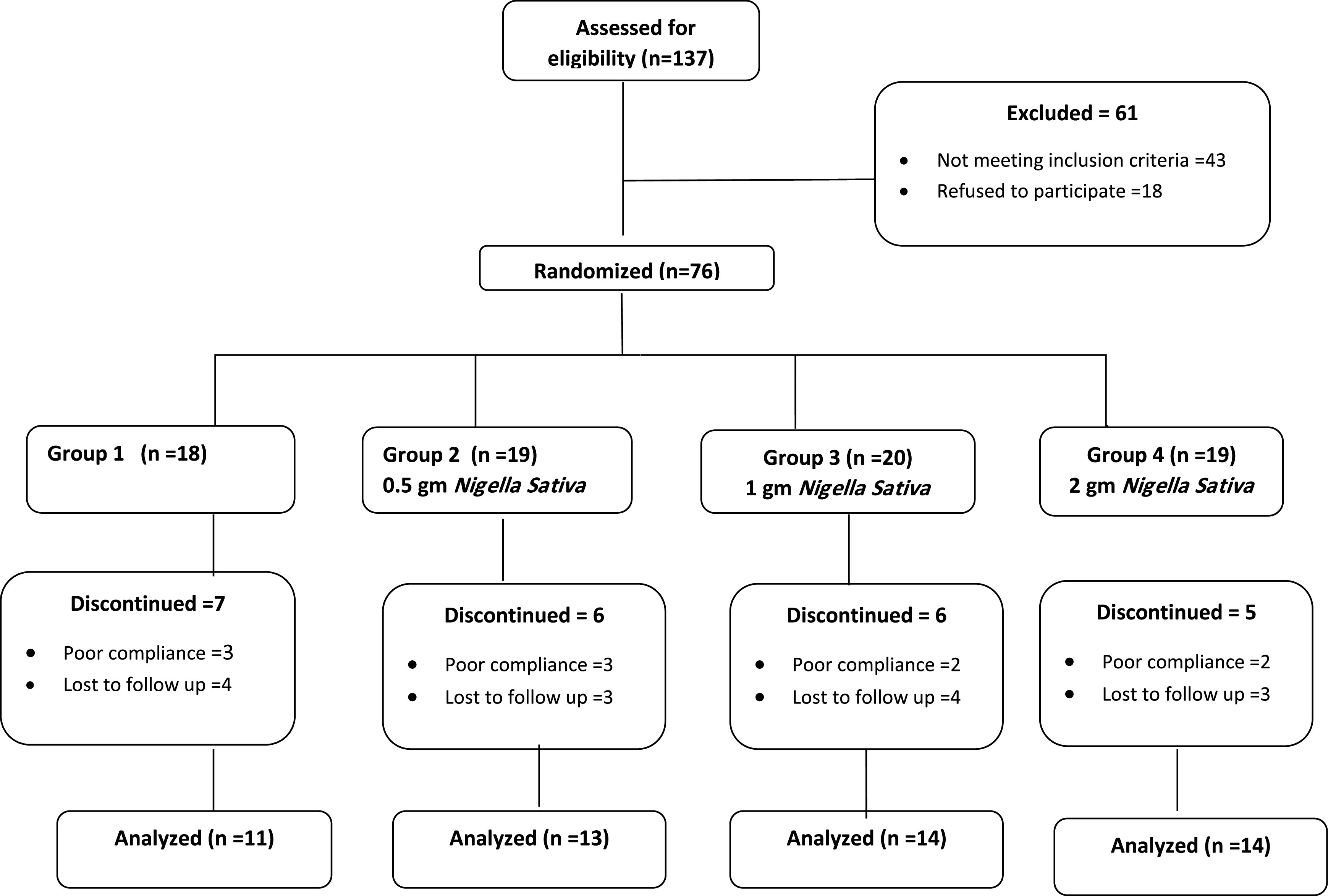
Study flow chart.

Participants were invited to join the study through a web page which included the consent form. The total number screened was 137 participants, 43 participants were excluded according to inclusion/exclusion criteria, and 18 refused to participate. Those fulfilling the criteria and agreed to participate were given appointments in the Family Medicine Center of Imam Abdulrahman bin Faisal University. 76 participants were enrolled in this study over three Sundays before intervention and three Sundays after intervention in the period February to March, 2020. 10 participants were excluded due to poor compliance (<90%), while 14 have withdrawn or lost to follow up. All participants tolerated the intervention, and no side effects were reported throughout the four weeks of treatment. Furthermore, all basic investigations including renal and liver function tests, and CBC were within normal limits. The study flow chart is shown in Figure 1. All groups were well matched in age, sex, BMI and other baseline characteristics as shown in Table 1.

**Table 1.** Demographic and baseline characteristics of the study groups.

Variables	Group 1	Group 2	Group 3	Group 4	*P*-value
Age	21.5333	22.2500	22.3529	20.8947	.023
Sex (M/F)	9/2	12/1	13/1	14/0	
BMI	25.2610	27.9479	28.7125	22.8367	.176
Lymphocytes absolute count 10 ^3^/μL	2.05 ± 0.48	1.91 ± 0.20	1.85 ± 0.24	2.03 ± 0.47	.851
T-lymphocytes (CD3+) cells/μL	1525.2 ± 333.21	1442 ± 217.79	1184.4 ± 75.60	1474.57 ± 299.50	.216
T helper cells (CD3+/CD4+) cells/μL	875.6 ± 186.52	808.5 ± 219.91	665.6 ± 141.66	861.86 ± 204.26	.283
T- suppressor cells (CD3+/CD8+) cells/μL	585 ± 243.6	502 ± 98.99	425.8 ± 77.62	562.71 ± 204.61	.540
B-lymphocytes (CD19+) cells/μL	256.4 ± 72.49	219 ± 49.49	268.8 ± 106.59	284.29 ± 109.42	.855
NK-cells (CD16+/CD56+) cells/μL	221.6 ± 100.15	210 ± 76.37	354.4 ± 163.17	237.57 ± 130.38	.348
CD4+/CD8+ ratio	1.79 ± 1.03	1.69 ± 0.77	1.64 ± 0.57	1.66 ± 0.55	.988
IL-1	21.95 ± 11.19	186.81 ± 172.51	51.72 ± 19.92	7.64 ± 1.64	.468
IL-4	Not detected				
IL-6	1022.14 ± 600.96	1903.52 ± 698.47	342.21 ± 153.72	1083.65 ± 411.25	.170
IL-10	Not detected				
IL-6 gene	27.28 ± 3.91	25.93 ± 2.16	27.25 ± 2.93	28.32 ± 3.51	.783
IL-13 gene	28.99 ± 3.50	30.13 ± 3.42	29.38 ± 3.10	31.62 ± 3.96	.748

### Heart rate and blood pressure

Vital signs were obtained from the participants before and after intervention; heart rate (HR), blood pressure (BP), and the mean arterial pressure (MAP). Group 2 participants showed a significant decrease in HR and systolic blood pressure from baselines (86.43 ± 18.48 beats/min *versus* 76.14 ± 11.35 beats/min, *P* < 0.05; and 130.15 ± 13.42 mmHg *versus* 119.69 ± 12.83 mmHg, *P* < 0.05), respectively. Moreover, group 3 showed a significant decrease in the diastolic blood pressure from 79.09 ± 7.46 to 66.09 ± 10.08 ( *P* < 0.05). No other significant changes were shown in HR, SBP, DBP, and the calculated MAP among groups ( Table 2).

**Table 2.** Effect of different doses of Nigella sativa on heart rate and blood pressure.

		Heart rate (bpm)	Systolic BP (mmHg)	Diastolic BP (mmHg)	MAP (mmHg)
Group 1 (Control)	B	74.75 ± 7.15	107.17 ± 53.55	59.17 ± 30.57	76.77 ± 37.89
	P	72.25 ± 6.24	122 ± 9.88	69.83 ± 6.08	87.22 ± 6.86
	n	4	6	6	6
	*P*-Value	0.504	0.535	0.456	0.533
Group 2 (0.5 g)	B	86.43 ± 18.48	130.15 ± 13.42	72.46 ± 10.45	86.56 ± 6.89
	P	76.14 ± 11.35	119.69 ± 12.83	69.23 ± 11.85	86.06 ± 10.52
	n	7	13	13	13
	*P*-Value	0.049	0.027	0.539	0.885
Group 3 (1 g)	B	89.17 ± 18.17	128.73 ± 8.87	79.09 ± 7.46	82.04 ± 28.37
	P	74.67 ± 14.76	125.36 ± 8.42	66.09 ± 10.08	85.85 ± 8.12
	n	6	11	11	11
	*P*-Value	0.136	0.327	0.009	0.701
Group 4 (2 g)	B	85.43 ± 7.39	124.5 ± 12.52	67.5 ± 9.68	83.43 ± 10.79
	P	89.71 ± 9.55	123.1 ± 10.08	71.9 ± 10.81	88.95 ± 8.18
	n	7	10	10	10
	*P*-Value	0.330	0.810	0.489	0.291

BP: Blood Pressure, MAP: Mean Arterial Pressure, B: Baseline, P: Post-Intervention, n: Sample Size.

### Lymphocyte count

Changes in lymphocyte profile are presented in Table 3. Lymphocyte absolute count and CD3+ lymphocytes were significantly increased in group 3, receiving 1 g *N. sativa*, compared with the baseline ( *P* < 0.05). T-helper cells (CD4+) were also significantly increased in group 3 ( *P* < 0.05). There was no significant change in other types of lymphocytes among all intervention groups.

**Table 3.** Effect of different doses of Nigella sativa on the lymphocytes profile.

		Lymphocytes absolute count 10 ^(3)^/μL	T-lymphocytes (CD3+) cells/μL	T helper cells (CD4+) cells/μL	T- suppressor cells (CD8+) cells/μL	B-lymphocytes (CD19+) cells/μL	NK-cells (CD16+/CD56+) cells/μL	CD4+/CD8+ ratio
Group 1 (Control)	B	2.05 ± 0.48	1525.2 ± 333.21	875.6 ± 186.52	585 ± 243.6	256.4 ± 72.49	221.6 ± 100.15	1.79 ± 1.03
	P	1.12 ± 0.64	1560.6 ± 426.15	893.4 ± 316.67	600.4 ± 260.85	258.8 ± 123.34	277.8 ± 74.12	1.81 ± 1.15
	n	5	5	5	5	5	5	5
	*P*-value	0.452	0.696	0.782	0.606	0.924	0.106	0.732
Group 2 (0.5 g)	B	1.91 ± 0.19	1442 ± 217.79	808.5 ± 219.91	502 ± 98.99	219 ± 49.49	210 ± 76.37	1.69 ± 0.77
	P	1.76 ± 0.2	1323.5 ± 44.54	702.5 ± 71.42	514.5 ± 166.17	166.5 ± 19.09	233.5 ± 161.93	1.47 ± 0.62
	n	2	2	2	2	2	2	2
	*P*-value	0.693	0.638	0.497	0.836	0.247	0.764	0.295
Group 3 (1 g)	B	1.85 ± 0.24	1184.4 ± 75.60	665.6 ± 141.66	425.8 ± 77.62	268.8 ± 106.59	354.4 ± 163.17	1.64 ± 0.57
	P	2.17 ± 0.26	1424.2 ± 114.51	841 ± 143.36	492.4 ± 59.19	340.6 ± 175.28	334.8 ± 212.06	1.74 ± 0.43
	n	5	5	5	5	5	5	5
	*P*-value	0.008	0.009	0.002	0.061	0.196	0.689	0.368
Group 4 (2 g)	B	1.91 ± 0.51	1371 ± 282.84	774.4 ± 139.74	560.4 ± 250.55	265.8 ± 128.23	240 ± 150.65	1.56 ± 0.62
	P	1.97 ± 0.33	1493.8 ± 188.09	887.8 ± 97.96	569.2 ± 160.67	274 ± 81.91	163.4 ± 73.15	1.67 ± 0.54
	n	5	5	5	5	5	5	5
	*P*-value	0.748	0.347	0.088	0.917	0.845	0.201	0.353

B = Baseline, P = Post-intervention, n = Sample Size.

### Immunoglobulin and cytokines

Immunoglobulins IgG and IgM showed non-significant changes in all intervention groups Table 4. The value of IL-4 and IL-10 were below the detection limits of the ELISA kits, the other cytokines (IL-1β, IL-6) did not show any significant changes in all groups ( Table 4).

**Table 4.** Effect of different doses of Nigella sativa on the levels of immunoglobulins and interleukins.

		IL-1 *Beta*	IL-4	IL-6	IL-10	IgG (mg/dL)	IgM (mg/dL)
Group 1 (Control)	B	21.95 ± 11.19	Not detected	1022.14 ± 600.96	Not detected	1400.2 ± 254.78	108 ± 254.78
	P	14.38 ± 11.06	Not detected	1486.68 ± 962.10	Not detected	1340.2 ± 173.03	107 ± 40.07
	n	3		8		5	5
	*P*-value	.275		.253		0.279	0.825
Group 2 (0.5 g)	B	186.81 ± 172.51	Not detected	1903.52 ± 698.47	Not detected	1361.5 ± 98.29	70.5 ± 17.68
	P	146.80 ± 140.28	Not detected	1906.96 ± 831.29	Not detected	1399 ± 107.48	74.5 ± 14.85
	n	3		13		2	2
	*P*-value	.341		.988		0.109	0.295
Group 3 (1 g)	B	51.72 ± 19.92	Not detected	342.21 ± 153.72	Not detected	1354.4 ± 317.42	126.8 ± 23.34
	P	48.60 ± 24.10	Not detected	351.15 ± 150.61	Not detected	1348.2 ± 329.69	126.8 ± 19.79
	n	4		9		5	5
	*P*-value	.731		.780		0.724	1
Group 4 (2 g)	B	7.64 ± 1.64	Not detected	1083.65 ± 411.25	Not detected	1080.2 ± 154.97	140.2 ± 115.66
	P	34.25 ± 17.23	Not detected	1353.25 ± 632.88	Not detected	1057.2 ± 130.02	140.8 ± 110.47
	n	7		12		5	5
	*P*-value	.159		.368		0.536	0.872

### Interleukin gene expression

**Figure 2.**
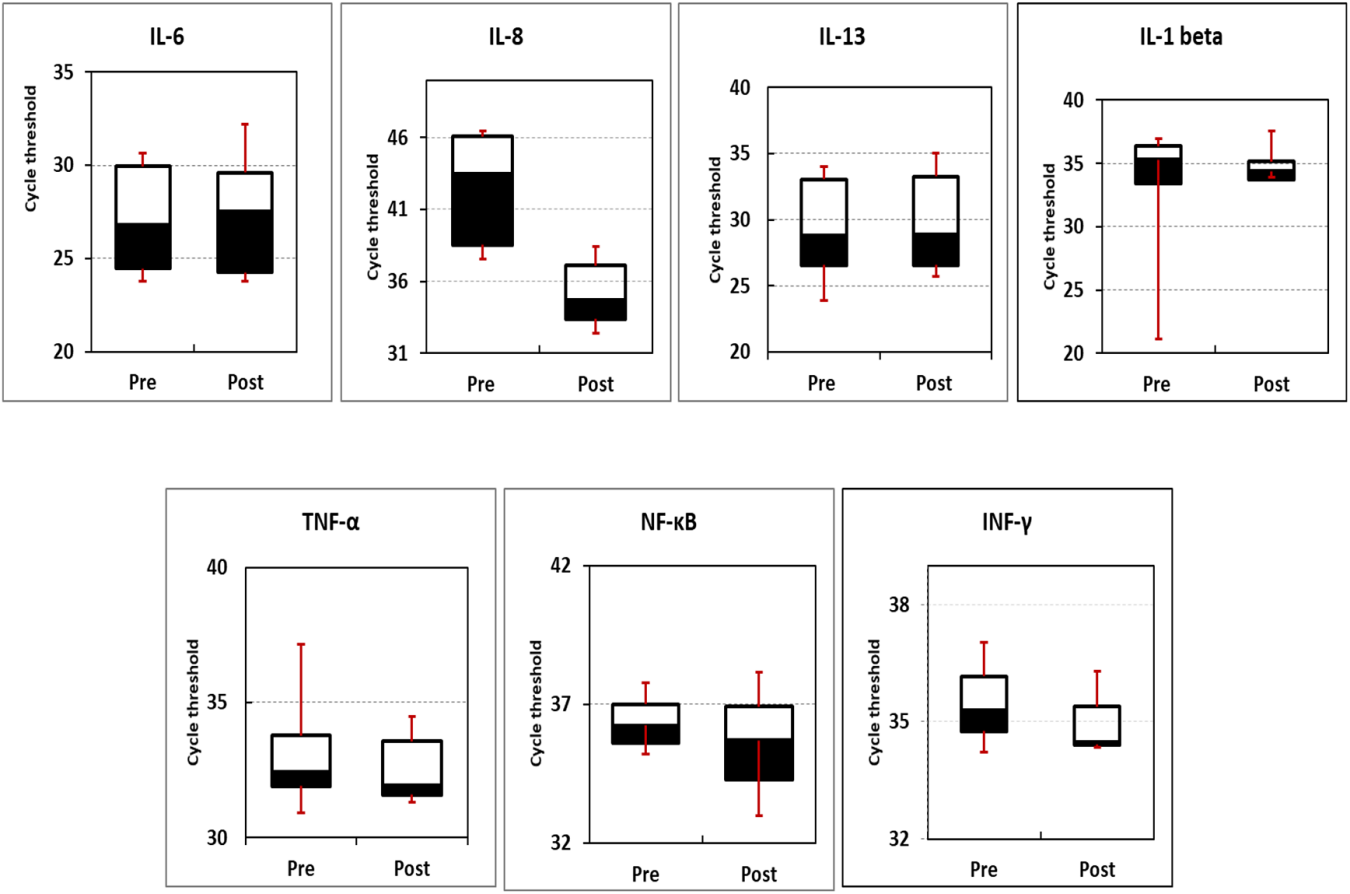
The box blotting of expression profile of the studied genes. Data indicated that there are no significant differences between pre- and post-intervention.

The expression profile of inflammation- and cancer-related genes (IL-1β, IL-8, TNF-α, IFN-γ, IL-6, NF-κB, and IL-13) were measured using qPCR. Box blotting ( Figure 2 and Table 5) showed no significant changes in the expression level of the studied genes in pre-intervention compared to post-intervention readings ( *P*-values of 0.15, 0.48, 0.15, 0.48, 0.37, 0.12, and 0.28 for IFN-γ, NF-κB, TNF-α, IL-1β, IL-13, IL-8, and IL-6, respectively).

**Table 5.** Effect of different doses of Nigella sativa on gene expressions.

		IL-1 *beta*-gene	IL-6 gene	IL-8 gene	Il-13 gene	TNF- *alpha*	NF- *Kappa-B*	INF- *gamma*
**Group 1 (Control)**	B	Not detected	27.28 ± 3.91	Not detected	28.99 ± 3.50	Not detected	35.85 ± 1.125	Not detected
	P	Not detected	28.84 ± 3.50	Not detected	33.44 ± 1.16	Not detected	34.05 ± .51	Not detected
	n		4		4		3	
	*P*-value		.410		.150		.171	
**Group 2 (0.5 g)**	B	Not detected	25.93 ± 2.16	Not detected	30.13 ± 3.42	Not detected	Not detected	Not detected
	P	Not detected	27.45 ± 3.06	Not detected	30.96 ± 2.80	Not detected	Not detected	Not detected
	n		4		4			
	*P*-value		.497		.741			
**Group 3 (1 g)**	B	Not detected	27.25 ± 2.93	Not detected	29.38 ± 3.10	Not detected	33.25 ± .004	Not detected
	P	Not detected	27.69 ± 3.37	Not detected	29.68 ± 3.74	Not detected	34.79 ± 1.503	Not detected
	n		6		7		2	
	*P*-value		.786		.865		.385	
**Group 4 (2 g)**	B	Not detected	28.32 ± 3.51	Not detected	31.62 ± 3.96	Not detected	Not detected	Not detected
	P	Not detected	28.96 ± 0.79	Not detected	31.68 ± 4.23	Not detected	Not detected	Not detected
	n		3		3			
	*P*-value		.802		.987			

B = Baseline, P = Post-intervention, n = Sample Size.

**Questionnaire:** Online questionnaire was distributed among the participants after the end of the study, the questionnaire contained seven questions, four of which were multiple choice questions, three answers were pre-set to: yes-positive effect, yes-negative effect and no change, and an option to add other observations. The control group had more infections during the period of the study in comparison to all test groups. More subjects reported a positive change in their daily activity in groups 3 and 4.

No participants of the control group noticed any change in their concentration (n = 15). On the other hand, 27.8% of the third group (n = 5) noted a positive change in their concentration. Most of the subjects in the control group (n = 14) did not notice any change in their sleep, while in Group 2, 12.5% (n = 2) noted a negative change in their sleep pattern. In groups with higher doses (Groups 3 and 4), more participants noted a positive change in their sleep pattern. The questionnaire results are showed in ( Table 6).

**Table 6.** Questionnaire.

	Answer	Did you get any infection during the time of the study?	Did you notice a change in your daily activity or energy?		Did you notice any change in your concentration?		Did you notice any change in your sleep pattern?	
			Positive change	Negative change	Positive change	Negative change	Positive change	Negative change
Group 1 (Control) n = 15	Yes	5 (33.3%)	4 (27%)	0%	0%	0%	1 (7%)	0%
	No	10 (66.7%)	11 (73%)		15 (100%)		14 (93%)	
Group 2 (0.5 g) n = 16	Yes	2 (12.5%)	5 (31%)	0%	4 (25%)	1 (6%)	2 (12.5%)	2 (12.5%)
	No	14 (87.5%)	11 (69%)		11 (69%)		12 (75%)	
Group 3 (1 g) n = 18	Yes	3 (17%)	7 (39%)	0%	5 (27.8%)	0%	4 (22%)	0%
	No	15 (83%)	11 (61%)		13 (72.3%)		14 (78%)	
Group 4 (2 g) n = 17	Yes	4 (24%)	6 (35%)	0%	2 (12%)	0%	4 (24%)	1 (5%)
	No	13 (76%)	11 (65%)		15 (88%)		13 (76%)	

## Discussion

The use of natural products and herbs as medicines is the practice of humans for centuries. One of the main mechanisms by which such herbs produce their beneficial effect on health is immunomodulation which involves stimulation or inhibition of the immune system. ^18^ Cardamom and black pepper are good examples of such herbs which possess a potent immunomodulatory effects. ^19^

*N. sativa*, commonly known as black seed or black cumin, is a very common herb which belongs to the Ranunculaceae family. ^20^^,^^21^ Several therapeutic effects have been attributed to *N. sativa* and its active ingredient, thymoquinone, including anti-histaminic, anti-inflammatory, anti-hypertensive, hypoglycemic, anti-cancer, and immunity-boosting effects ^21^^,^^22^ The immunoregulatory effect of *N. sativa* has been studied in animals and several positive potential effects in enhancing immunity have been reported. However, the current clinical trial is the first to study the immunomodulatory effect of *N. sativa* on healthy humans, and the first to investigate its optimal dose.

Our results showed a significant increase of T-helper cells (CD4+), this is in agreement with a study conducted on beta-thalassemia major children where *N. sativa* enhanced the cell-mediated immunity significantly *via* increasing CD4 counts (from 1319.88 ± 74.56 to 2007.64 ± 90.34 cells μL ^−1^) ( *P* < 0.001). ^23^ This study also reported a significant increase in T-suppressor cells from 727.09 ± 42.81 cells μL ^−1^ to 1145.31 ± 77.58 cells μL ^−1^ after *N. sativa* intervention, ( *P* < 0.001). However, this parameter was non-significantly increased in our study from 425.8 ±77.62 cells μL ^−1^ to 492.4 ± 59.19 cells μL ^−1^ after giving 1 g of *N. sativa* for 1 month ( *P* = 0.061). ^23^ Furthermore, CD4+ (helper) T lymphocytes has been reported to be stimulated by *N. sativa* oil in a murine cytomegalovirus (CMV) model using BALB/c mice. ^24^

Increase in absolute lymphocyte count was also shown in our study, this is supported by a study conducted on diabetic hamsters, where oral administration of *N. sativa* oil improved lymphocyte count in streptozotocin (STZ)-induced diabetic hamsters. ^12^ Additionally, oral administration of *N. sativa* oil significantly increased peripheral lymphocyte and monocyte counts in antigen-challenged rats. ^25^

Oral administration of *N. sativa* oil (90 mg·kg ^−1^ per day) for 30–60 days elevated neutrophils and lymphocytes back to normal levels in chloramphenicol treated Albino rats. ^26^ *N. sativa* seeds extract induced a stimulatory effect on unactivated lymphocytes cell culture. ^13^

In our study, the increase in the previously discussed cells has been lost when the dose was increased from 1 g *N. sativa* to 2 g, which is consistent with the results observed in previous two different studies. One study was on the effect of *N. sativa* on *Helicobacter pylori* eradication, where 2 g *N. sativa* shown to be more effective than 1 g *N. sativa* and 3 g *N. sativa* in comparison to triple therapy. ^17^ The other study was conducted on the effect of *N. sativa* on the glycemic control in type 2 diabetic patients which showed that 2 g *N. sativa* was more effective than the 3-g dose in reducing fasting blood glucose, 2-hour post-load glucose and hemoglobin A1C. ^16^

The current study showed a non-significant effect of *N. sativa* on IgG and IgM. Previous studies on the effect of *N. sativa* on humoral immunity showed inconsistent results. Sapmaz *et al*., reported that *N. sativa* oil produced a significant decrease in serum complement component 3 (C3), IgM and IgA levels with no significant change IgG level in rats, treated with formaldehyde inhalation. ^27^ Moreover, a study of the effect of thymoquinone (TQ), which is the active ingredient of *N. sativa*, was conducted on rats and showed increase in total levels of immunoglobulins (IgG and IgM) and antibody hemagglutination in TQ-supplemented group. ^28^ In contrast, oral administration of *N. sativa* extract and TQ in mice with allergic diarrhea did not produce a significant effect on total IgE level or ovalbumin-specific IgE. ^29^ These discrepancies in the *N. sativa* effect on immunoglobulins may be better explained by the differences in species and/or condition of the species among various studies.

The current study did not show any significant effect of *N. sativa* on the level of IL-1β and IL-6. Similar findings were reported by Duncker *et al*., where no significant change was shown in IL-4, IL-5, IL-10 or IFN-γ secretion by mesenteric lymphocytes in mice treated with both oral extract of *N. sativa*, and intra-gastric TQ. ^29^ Another study showed that *N. sativa* extracts had no effect on the secretion of IL-4 and IL-2 from lymphocytes, both in presence and absence of PWM. ^13^ In contrast, significant increase in IL-10, but not TNFα was observed after 8 weeks of oral administration of *N. sativa* oil (1 g per patient per day) in rheumatoid arthritis patients. ^30^ Furthermore, *N. sativa* extracts increased the secretion of IL-3 from PBMCs cultured in presence or absence of pooled allogeneic cells. ^13^ Theses discrepancies in the effect of *N. sativa* on various cytokines could be explained by differences in the preparations ( *i.e.,* extracts, oil, *etc.*), doses of *N. sativa*, study designs ( *i.e., in vitro* or *in vivo*), species used (other animals *versus* human), and difference in the studied cell/animal condition.

### Effect on BP and heart rate

Our results showed that the second group (0.5 g *N. sativa*) and the third group (1 g *N. sativa*) had a significant reduction in in the systolic BP for group 2 and the diastolic BP for group 3 ( *P* < 0.05), respectively. This result is supported by a randomized double-blinded placebo-controlled trial conducted by Huseini and others in 2013 on healthy adult volunteers, where 5 mL of *N. sativa* oil was administered to 70 participants for an 8-week period. The study resulted in, average of 8.17% decrease in the systolic BP and 12.46% decrease in the diastolic BP. ^31^

### Gene expression profiling

In the present investigation, the expression profiles of seven genes were evaluated before and after intervention with *N. sativa.* Box blotting of the data showed no significant variation in the expression profiles of these genes either amongst them or before and after treatment within the same gene ( *P*-values of 0.15, 0.48, 0.15, 0.48, 0.37, 0.12, and 0.28 for IFN-γ, NF-κB, TNF-α, IL-1β, IL-13, IL-8, and IL-6, respectively).

The action of *N. sativa* and its ingredients on cytokines and inflammatory markers varies depending on the cell or animal tested and its condition. For example, herbal melanin, extracted from *N. sativa*, enhanced the production of m-RNA expression of TNF-α, and IL-6 in normal human peripheral blood mononuclear cells. ^32^ While, it has been suggested that the treatment with TQ inhibited the production of TNF-α-induced IL-6 and IL-8 in rheumatoid arthritis synovial fibroblasts. ^33^

Furthermore, the therapeutic effects of alpha-hederin extracted from *N. sativa* has been extensively studied in terms of lung inflammation in rats. Treated animals showed elevated levels of IL-13 compared with the control group. These data indicated that alpha-hederin, like TQ, can indirectly intervene with IL-13 to reduce the inflammatory response. ^34^

On the other hand, oral administration of *N. sativa* oil reduced the level of different cytokines including IL-13. ^35^ Furthermore, TQ was found to inhibit NF-κB, TNF-α, IL-1β, and IL-6 induced by CLP. ^36^

The dual action of *N. sativa* has been suggested by other research groups; it upregulates IL-8 in non-activated PBMC cells and downregulates it in PWM-activated PBMC cells. ^13^ TQ also can ameliorate the toxic effects of arsenic *via* downregulating TNF-α and IFN-γ when administered three days before exposure to arsenic, ^37^ and reduced levels of NF-κB and TNF-α. ^38^

Nonetheless, this study shows no effect of a one-month *N. sativa* supplementation in young healthy human on the studied gene expressions and calls for further investigation on other immunity related genes and molecular mechanisms.

## Conclusion

Our results suggest that *N. sativa* has an immunopotentiation effect; the optimum dose seems to be 1 g for young healthy subjects. We recommend more future clinical trials to explore the use of *N. sativa* as an immune enhancer for various age groups in different health conditions.

### Limitations

1. Participants’ withdrawal due to the coronavirus disease 2019 (COVID-19) crisis.
2. Post-intervention results of certain interleukins and CD profile could not be obtained for many participants due to the COVID-19 crisis.

## Data availability

### Underlying data

Dryad: Underlying data for ‘Effect of *Nigella sativa* on general health and immune system in young healthy volunteers; a randomized, placebo-controlled, double-blinded clinical trial’. https://doi.org/10.5061/dryad.00000004b ^39^

Data are available under the terms of the Creative Commons Zero “No rights reserved” data waiver (CC0 1.0 Public domain dedication).
