# Supplementary material for: Effect of Nigella sativa on general health and immune system in young healthy volunteers; a randomized, placebo-controlled, double-blinded clinical trial
Source: F1000Res. 2023 Oct 18;10:1199. Originally published 2021 Nov 25. [Version 2] doi: 10.12688/f1000research.73524.2 (PMC10600512; doi:10.12688/f1000research.73524.2)
Supplement: Peer review history

# Reviewer report: Reviewer response for version 2

**DOI:** 10.5256/f1000research.157503.r216596

**Published:** 2023-10-25

**Authors:** Siddig Ibrahim Abdelwahab

**Affiliations:** 1 Medical Research Center, Jazan University, Jazan, Jazan, Saudi Arabia

I am fine with changes. All are OK

Is the work clearly and accurately presented and does it cite the current literature?

Yes

If applicable, is the statistical analysis and its interpretation appropriate?

Yes

Are all the source data underlying the results available to ensure full reproducibility?

Partly

Is the study design appropriate and is the work technically sound?

Yes

Are the conclusions drawn adequately supported by the results?

Yes

Are sufficient details of methods and analysis provided to allow replication by others?

Yes

Reviewer Expertise:

NA

I confirm that I have read this submission and believe that I have an appropriate level of expertise to confirm that it is of an acceptable scientific standard.

# Reviewer report: Reviewer response for version 2

**DOI:** 10.5256/f1000research.157503.r216595

**Published:** 2023-10-23

**Authors:** Mohammad Akram Randhawa

**Affiliations:** 1 Department of Pharmacology, College of Medicine, Northern Border University, Arar, Saudi Arabia

I have read the revised version of the manuscript. **I believe the authors have made appropriate additions in response to my earlier comments. I really appreciate their efforts.

Is the work clearly and accurately presented and does it cite the current literature?

Yes

If applicable, is the statistical analysis and its interpretation appropriate?

Yes

Are all the source data underlying the results available to ensure full reproducibility?

Yes

Is the study design appropriate and is the work technically sound?

Yes

Are the conclusions drawn adequately supported by the results?

Yes

Are sufficient details of methods and analysis provided to allow replication by others?

Yes

Reviewer Expertise:

NA

I confirm that I have read this submission and believe that I have an appropriate level of expertise to confirm that it is of an acceptable scientific standard.

# Reviewer report: Reviewer response for version 1

**DOI:** 10.5256/f1000research.77179.r208614

**Published:** 2023-10-11

**Authors:** Siddig Ibrahim Abdelwahab

**Affiliations:** 1 Medical Research Center, Jazan University, Jazan, Jazan, Saudi Arabia

- Limited sample size and gender imbalance: The study includes only 52 healthy subjects, with a significant gender imbalance (48 males and 4 females). This small sample size and gender disparity may limit the generalizability of the findings and raise concerns about the representativeness of the population.
- Lack of long-term follow-up: The trial's duration is only four weeks, which may not be sufficient to capture the full range of effects on immune parameters. Longer-term follow-up would provide a better understanding of the sustained effects and potential adverse reactions associated with N. sativa supplementation.

In the introduction, kindly correct according to following suggestions:

1. "Nigella sativa is one of the most commonly used herbal medicines worldwide nowadays."
2. "It has been used for more than 2000 years as a natural remedy for various illnesses."
3. "Research has documented its therapeutic potential as an antimicrobial, anti-inflammatory, antioxidant, antidiabetic, antihypertensive, antitumor, and immunomodulatory agent [1,2]."
4. "The immune system consists of multiple interconnected networks of cells, proteins, and lymphoid organs that provide protection against millions of microbes and infections."
5. "The immune system includes innate immunity and adaptive immunity. Innate immunity provides immediate protection to the body, and its effect is similar in different individuals, while adaptive immunity takes more time to develop and is more specific and effective against invasive pathogens [3]."
6. "The immunomodulatory effect of N. sativa has been extensively studied on both innate and adaptive immunity, as well as related messengers and mediators [4–6]."
7. "Several in vivo studies have shown significant effects of N. sativa on immunity, autoimmune diseases, and toxicity."
8. "A study demonstrated that the aqueous extract of N. sativa resulted in a significant increase (62.3% ± 6.4%) in natural killer (NK) cytotoxic cell activity against YAC-1 cells after 1 week of oral administration in 10-week-old BALB/c female mice [7]."
9. "Another in vitro study on the effect of thymoquinone (TQ) on immunity showed that TQ injected directly into cells at low concentrations (10, 2.5, or 0.62 μg mL−1) increased the survival of activated T-cells and the ability of CD8+ T-cells to generate IFN-γ, indicating the potential benefits of TQ against infectious diseases and immune enhancement [8]."
10. "N. sativa supplementation at levels of 4% or 5% positively enhanced immunity against Newcastle disease virus in newly evolved crossbred laying hens [9]."
11. "In another study on Newcastle virus vaccinated broilers, N. sativa supplementation at three doses (5, 10, and 20 g kg−1) for 42 days significantly increased antibodies against Newcastle virus on day 35 [10]."
12. "A diet supplemented with 40 g kg−1 N. sativa fed to broiler chicks improved antibody production against both Newcastle virus and infectious bursal disease [11]."
13. "The phagocytic index and rate were significantly higher in STZ-diabetic hamsters treated with N. sativa oil (NSO) at a dose of 400 mg·kg−1 for 4 weeks compared to untreated diabetic animals, as demonstrated by fluorescence microscopy [12]."
14. "N. sativa extract has a stimulatory effect on cellular immunity in vitro by increasing the proliferative capacity of T lymphocytes and splenocytes, as well as the response to different mitogens of human peripheral blood mononuclear cells (PBMC) [13]."
15. "NSO was shown to possess a protective role against vitamin A hypervitaminosis. Rats treated orally with 800 mg·kg−1 NSO showed higher serum levels of IgG and IgM than the control group or those receiving high doses of vitamin A [14]."
16. "Gestational diabetes rats showed improvement in the immune status of their offspring after oral antenatal feeding with 20 mg kg−1 TQ, reversing the decreased levels of IL-2, T-cell reproduction, and improving both circulating and thymus-homing T-cell proliferation [15]."
17. "The literature above demonstrates a very promising immunomodulating effect of N. sativa."
18. "However, the immunopotentiation effect of this remarkable plant has not been investigated in normal humans."
19. "Hence, this study was designed to evaluate the impact of different doses of N. sativa on the immune system in young, healthy humans."

The conclusion could be improved by providing more specific details, addressing the limitations of the study, suggesting specific research questions for future trials, considering the generalizability of the findings, and discussing potential risks or side effects associated with the use of N. sativa.

Is the work clearly and accurately presented and does it cite the current literature?

Yes

If applicable, is the statistical analysis and its interpretation appropriate?

Yes

Are all the source data underlying the results available to ensure full reproducibility?

Partly

Is the study design appropriate and is the work technically sound?

Yes

Are the conclusions drawn adequately supported by the results?

Yes

Are sufficient details of methods and analysis provided to allow replication by others?

Yes

Reviewer Expertise:

Pharmacology

I confirm that I have read this submission and believe that I have an appropriate level of expertise to confirm that it is of an acceptable scientific standard, however I have significant reservations, as outlined above.

# Reviewer report: Reviewer response for version 1

**DOI:** 10.5256/f1000research.77179.r101149

**Published:** 2022-01-18

**Authors:** Mohammad Akram Randhawa

**Affiliations:** 1 Department of Pharmacology, College of Medicine, Northern Border University, Arar, Saudi Arabia

The study is well designed and conducted a placebo controlled double blind clinical trial that confirms the immunopotentiation effect of *Nigella sativa* powder, manifested as elevation of helper T cells, in young healthy volunteers. Surprisingly, 2 g *N. sativa* once daily for one month failed to show a similar increase in helper T-cells than with 1g. To make the article scientifically sound the authors should discuss the possible reasons for that.

 Perhaps, the higher doses trigger some inhibitory mechanisms. The evidence in favour is that the *N. sativa* & thymoquinone have been shown in the literature to possess beneficial effects in rheumatoid arthritis and asthma by suppressing some immune mechanisms. A careful search of the literature would reveal more such examples and if we calculate dose/concentration used in these cases per 70 kg (Adult human dose), perhaps, would be equal to or higher than 2g *N. sativa* powder per day.

 It is desired that the authors find such examples (Human or animals studies) and describe the possible reasons for the absence of elevation in helper T cells by increasing the dose of *N. sativa* to 2g.

 Some other minor alterations have been suggested in the text (see attached and annotated report).

Is the work clearly and accurately presented and does it cite the current literature?

Yes

If applicable, is the statistical analysis and its interpretation appropriate?

Yes

Are all the source data underlying the results available to ensure full reproducibility?

Yes

Is the study design appropriate and is the work technically sound?

Yes

Are the conclusions drawn adequately supported by the results?

Yes

Are sufficient details of methods and analysis provided to allow replication by others?

Yes

Reviewer Expertise:

NA

I confirm that I have read this submission and believe that I have an appropriate level of expertise to confirm that it is of an acceptable scientific standard, however I have significant reservations, as outlined above.

# Response

**Published:** 2022-01-26

**Authors:** Abdullah Bamosa

**Affiliations:** Imam Abdulrahman bin Faisal univeristy, Saudi Arabia

We would like to thank Professor Akram for his valuable comments. We have actually discussed the point of losing the significant increase in helper T cells when *Nigella sativa* dose was increased from 1 to 2g as we have encountered such decrease in the effect of *Nigella sativa *when increasing the dose in previous clinical trials on diabetic patients and H pylori eradication in dyspeptic patients.

 We agree that the most possible cause is the presence of some ingredients that possess negative effect at higher doses. *Nigella sativa* has several active ingredients which makes such explanation sound and logical. Our trial was on young healthy subjects expected to have higher response to lower doses than old or ill subjects. This fact supports our result of higher effect by 1 g than 2 g dose of Nigella sativa and based on that we concluded that optimum dose for enhancing immunity in this age group seems to be 1 g of Nigella sativa.
